# Pain and its Perceived Relatedness to the Onset, Maintenance, and Relapse of Opioid use Disorder: A Descriptive Study of Non-Treatment-Seeking Individuals

**DOI:** 10.1080/24740527.2024.2332198

**Published:** 2024-03-20

**Authors:** Johnathan Rausch, Parker Entrup, Megan Deaner, Jasmine King, O. Trent Hall

**Affiliations:** a Department of Psychiatry and Behavioral Health, Ohio State University Wexner Medical Center Talbot Hall, Columbus, OH, USA; b College of Medicine, The Ohio State University, Columbus, OH, USA

**Keywords:** Addiction medicine, chronic pain, substance misuse

## Abstract

**Background:**

Previous research has found chronic pain to be prevalent among individuals with opioid use disorder (OUD). The perception that pain is related to OUD onset, maintenance, relapse, and treatment delay has been noted in this population. However, prior works primarily involved treatment-engaged populations. Scant research describes such perceptions among non-treatment-seeking individuals.

**Aims:**

This study describes pain burden and perceptions regarding the role of pain in OUD onset, maintenance, relapse, and addiction treatment delay in a sample of individuals with untreated OUD.

**Methods:**

This cross-sectional study surveyed syringe exchange participants (*n* = 141). Participants responded to a survey including *Diagnostic and Statistical Manual of Mental Disorders*, Fifth Edition OUD criteria, pain survey scales, demographic characteristics, and questions regarding pain and its perceived relatedness to aspects of OUD.

**Results:**

Most participants reported pain within the past 4 weeks (127, 91.4%). Data displayed a skew toward more intense pain ratings, with 120 reporting their pain as greater than mild (86.3%). A majority of participants agreed that pain was responsible for their OUD onset (79, 56.4%), maintenance (76, 54.3%), past relapse experience (82, 57.9%), and treatment delay (81, 57.9%). Correlative analyses revealed that pain severity and interference measures displayed moderate and statistically significant associations with extent of perceived relatedness of pain to these aspects of OUD.

**Conclusions:**

Among this sample of individuals with untreated OUD, pain and pain interference were prevalent. Pain was perceived to be related to OUD onset, maintenance, relapse, and treatment delay by a majority of the sample. These findings are in accordance with and expand upon prior works.

**Abbreviations:**

OUD: opioid use disorder; DSM-5: Diagnostic and Statistical Manual 5; BPI: Brief Pain Inventory; NIDA: National Institute on Drug Abuse; IASP: International Association for the Study of Pain; MOUD: Medications for Opioid Use Disorder; IQR: Interquartile Range.

## Résumé

**Contexte**: Des recherches antérieures ont montré que la douleur chronique est prévalente chez les personnes souffrant d’un trouble lié à l’utilisation d’opioïdes (TUO). La perception que la douleur est liée à l’apparition, au maintien, à la rechute et au retard dans le début du traitement a été constatée au sein de cette population. Toutefois, les travaux antérieurs portaient principalement sur les populations engagées dans un traitement. Peu de recherches décrivent ces perceptions chez les personnes qui ne sont pas à la recherche d’un traitement.

**Objectifs:** Cette étude décrit le fardeau de la douleur et les perceptions concernant le rôle de la douleur dans l’apparition, le maintien, la rechute et le retard dans le début du traitement du trouble lié à l’utilisation d’opioïdes chez un échantillon de personnes souffrant d’un trouble lié à l’utilisation d’opioïdes non traité.

**Méthodes:** Cette étude transversale a interrogé des participants pratiquant l’échange de seringues (n = 141). Les participants ont répondu à un questionnaire comprenant les critères de la cinquième édition du Manuel diagnostique et statistique des troubles mentaux pour le trouble lié à l’utilisation d’opioïdes, des échelles d’évaluation de la douleur, leurs caractéristiques démographiques, ainsi que des questions concernant la douleur et son lien perçu avec différents aspects du trouble lié à l’utilisation d’opioïdes.

**Résultats:** La plupart des participants ont déclaré avoir ressenti de la douleur au cours des quatre dernières semaines (127, 91,4 %). Les données ont montré une tendance à évaluer la douleur de façon plus intense, 120 participants ayant déclaré que leur douleur était plus que légère (86,3 %). La majorité des participants ont reconnu que la douleur était responsable de l’apparition (79, 56,4 %), du maintien (76, 54,3 %), des rechutes antérieures (82, 57,9 %) et du retard dans le début du traitement (81, 57,9 %) du trouble lié à l’utilisation des opioïdes. Les analyses corrélatives ont révélé que les mesures de l’intensité et de l’interférence de la douleur présentaient des associations modérées et statistiquement significatives avec l’étendue du lien perçu entre la douleur et ces aspects du trouble lié à l’utilisation des opioïdes.

**Conclusions:** Dans cet échantillon de personnes atteintes d’un trouble lié à l’utilisation des opioïdes non traité, la douleur et l’interférence de la douleur étaient prévalentes. La douleur était perçue comme étant liée à l’apparition, au maintien, à la rechute et au retard dans le début du traitement du trouble lié à l’utilisation des opioïdes par la majeure partie de l’échantillon. Ces résultats sont conformes aux travaux antérieurs et les étayent.

**Abréviations:** TUO : trouble lié à l’utilisation d’opioïdes; DSM-5 : Manuel diagnostique et statistique 5; BPI : Questionnaire concis sur la douleur; NIDA : National Institute on Drug Abuse; IASP : Association internationale pour l’étude de la douleur; MTUO : Médicaments pour le trouble lié à l’utilisation des opioïdes; EIQ : Écart interquartile.

## Introduction

According to the International Association for the Study of Pain, pain is “an unpleasant sensory and emotional experience associated with, or resembling that associated with, actual or potential tissue damage.”^1^ As an aversive personal experience, pain may set the stage for negative reinforcement processes implicated in the development and maintenance of addiction.^2–4^ Increased understanding of how pain functions in untreated opioid use disorder (OUD) is critical, because it may lead to more targeted engagement strategies to reach non-treatment-seeking individuals with pain and OUD.

Prior research has investigated how treatment-engaged people with OUD perceive pain and its relationship to their OUD. Pain has been identified as a theme in studies asking patients with OUD about how their OUD began or why they might have delayed accessing OUD treatment.^5–7^ Recently, our group described such perceptions among treatment-engaged individuals with OUD and yielded evidence of substantial pain burden and prevalent perceptions that pain was related to the onset, maintenance, and relapse of OUD.^8,9^ However, such work only involved participants who were engaged in treatment. Given that the vast majority of people living with OUD are untreated, further assessment of the non-treatment-seeking population is needed.^10^ Minimal research has investigated how non-treatment-seeking people with OUD perceive pain and its potential relationship to their addiction. One previous study found elevated pain prevalence, pain severity, and pain interference between non-treatment-seeking individuals with OUD relative to controls without OUD.^11^ That study also noted that participants reported pain as a motive for their opioid use. However, this prior study only investigated individuals misusing prescription opioid analgesics and did not probe relationships between these pain measures with specific aspects of OUD such as onset, maintenance, relapse, or treatment delay. Therefore, the present study aimed to describe pain burden (prevalence, severity, and interference) and assess pain’s perceived relatedness to OUD onset, maintenance, relapse, and treatment delay among non-treatment-seeking individuals with OUD who have a more diverse pattern of opioid use.

## Materials and Methods

### Participants

Participants (*n* = 153) were adult clients of a syringe exchange who were not currently engaged in substance use treatment per self-report. The present study did not assess treatment history but only current treatment status. This study investigates those who endorsed opioid use in the past 7 days and met *Diagnostic and Statistical Manual of Mental Disorders*, Fifth Edition (DSM-5) OUD criteria per self-report. One individual did not meet OUD criteria, six denied opioid use in the last 7 days, and five provided no answer regarding opioid use in the last 7 days. Thus, the final sample size was 141.

### Measures

The survey included demographic information, a 6-point Likert scale on pain severity over the past 4 weeks modeled after the RAND 36-Item Health Survey item 21, questions regarding sites of chronic pain (pain lasting longer than 3 months), Brief Pain Inventory (BPI) Pain Interference subscale, original questions about pain and OUD, and a DSM-5 OUD self-report form designed by the National Institute on Drug Abuse.^12–14^ Original questions involved having participants rate their level of agreement with statements regarding pain’s relatedness to aspects of OUD using a 5-point Likert scale with the options *strongly disagree, disagree, neutral, agree*, or *strongly agree*. These aspects were onset, maintenance, relapse, and treatment delay. In the survey, these items were phrased in terms of pain having been a motivator for first starting opioid use, continuing to use opioids, a past relapse experience, and putting off treatment due to fear of pain worsening, respectively.

The BPI Pain Interference subscale items are scored 0 to 10 from *does not interfere* to *completely interferes* and summed to a score out of 70. The scale can also be further divided into two dimensions: an affective dimension (sleep, relationships, life enjoyment, and mood) scored out of 40 and an activity dimension (general activity, normal work, and walking) scored out of 30.^13,15^ Though there is overlap between dimensions, there is proposed value in addressing these dimensions separately, because each suggests a different potential target for intervention.^8,16^ Participants were given instructions and assisted with navigating the survey as needed but otherwise completed the survey on their own using secure tablet computers. Survey data were collected using REDCap, a secure web platform for collecting and storing personal health information and managing online databases.^17^

### Data Analysis

Descriptive analyses (central tendency, frequency, and percentage) were used to analyze sample demographic characteristics, characteristics related to opioid use (such as OUD severity, times of opioid use per day, and routes of administration), as well as prevalence of pain and agreement with pain-related perceptions regarding aspects of OUD. Internal reliability of the BPI Pain Interference subscale, along with its activity and affective dimensions, was assessed using Cronbach’s alpha. Correlative analyses were performed between pain variables and level of agreement with perceptions that pain was related to OUD onset, maintenance, relapse, and treatment delay. The correlative analyses were all conducted using a two-tailed Spearman’s rho nonparametric partial correlation. This method does not depend on assumptions of normality or associative linearity and allows specified confounders to be mathematically controlled for.^18,19^ For all correlations, age and gender were controlled for and the size of *r_s_* was interpreted as [0.1, 0.3] = weak association, [0.3, 0.5] = moderate association, [0.5, 1] = strong association.^20^ Fisher’s *Z* transformation analyses were used to assess any statistically significant difference between the two subdomains of pain interference with respect to any correlation run against pain interference totals. All statistical analyses were conducted with SPSS v28.0.^21^

### Ethics

This cross-sectional survey was approved by the Ohio State University Wexner Medical Center Institutional Review Board (Approval No. 2022H0369) and conducted by trained Ohio State University Wexner Medical Center Talbot Hall staff at a syringe exchange in Columbus, Ohio, from January 10, 2023, to April 12, 2023. Participants provided verbal consent and were monetarily compensated with either bus passes or a visa gift card, each with a value of US$15.

## Results

### Sample Characteristics

All 141 participants responded to at least one or more of the following items: age of first opioid use, OUD DSM criteria, route of administration, and number of times they used opioids per day. Nearly all participants met the criteria for severe OUD (94.3%; six or more DSM-5 OUD criteria), and most reported opioid use three or more times daily (88.7%). The most reported route of administration was intravenous (83.7%). Complete sample characteristics are summarized in Table 1.

**Table 1.** Sample characteristics.

Characteristic	Participants (*n* =141)	Valid *n*
Age of first opioid use, mean (SD)		139
Years	23.91 (9.3)	
OUD severity, mean (SD)		141
DSM-5 criteria met	9.8 (1.9)	
Route of administration, *n* (%)		141
Intravenous	118 (83.7)	
Intramuscular or subcutaneous	12 (8.5)	
Smoking	69 (48.9)	
Snorting/sniffing up the nose	37 (26.2)	
Swallowing by mouth	12 (8.5)	
Another route	2 (1.4)	
Number of times opioids used per day, mean (SD)		141
Times used per day	4.02 (1.16)	
Age at time of survey, mean (SD)		140
Years	37.7 (8.1)	
Gender, *n* (%)		137
Woman, cisgender	58 (41.4)	
Man, cisgender	75 (53.6)	
Woman, transgender	1 (0.7)	
Unlisted or prefer not to say	3 (2.1)	
Racial identity, *n* (%)		141
Black or African American	17 (12.1)	
White or Caucasian	114 (80.9)	
Native American or Alaskan	4 (2.8)	
Prefer not to say	6 (4.3)	
Ethnicity, *n* (%)		137
Hispanic	2 (1.4)	
Non-Hispanic	123 (87.9)	
Prefer not to say	12 (8.6)	

One participant did not provide demographic information but did provide data for OUD-related variables. Four participants were missing data for gender and ethnicity; this includes the individual who did not provide any demographic information. Two participants were missing data for age of first opioid use. For route of administration, participants could choose more than one option. Percentages are calculated out of the overall sample (*n* = 141). However, a valid *n* column is provided for clarity on missing data for each variable.

### Pain and OUD

Pain was prevalent in the sample. One hundred twelve participants reported chronic pain in one or more body regions (80.0%), with the lower back being the most frequent site of pain (*n* = 88, 62.4%). Over 91% reported bodily pain within the past 4 weeks (*n* = 127, 91.4%), with a majority reporting their pain as greater than mild; that is, moderate, severe, or very severe (*n* = 120, 86.3%). Pain interference was also common among participants. Overall pain interference, as well as the affective dimension, demonstrated excellent internal reliability in this sample with Cronbach’s alpha coefficients of 0.93 and 0.91, respectively. The activity dimension of pain interference demonstrated good internal reliability with a Cronbach’s alpha of 0.869. BPI Pain Interference total score and the Affective and Activity subscales were not normally distributed as assessed by Shapiro-Wilk tests (*P* < 0.05). The total pain interference median was 42.5 (interquartile range [IQR] = 23.3–56) out of 70. The affective interference median was 26.0 (IQR = 14–33) out of 40, and the activity interference median was 16.5 (IQR = 7.3–23.0) out of 30.

**Figure 1.**
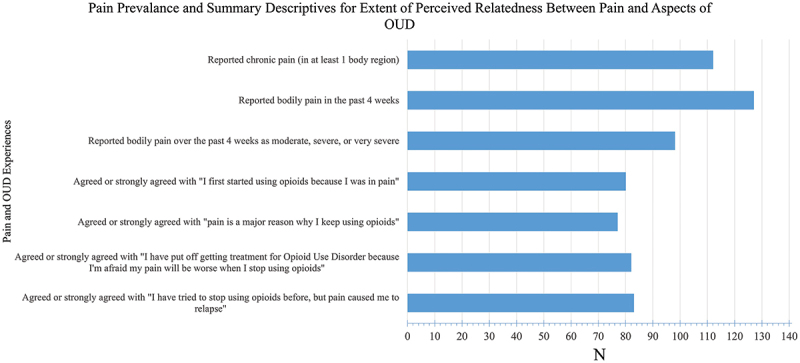
Pain prevalence and summary descriptives for extent of perceived relatedness between pain and aspects of OUD.

With respect to the perceived relatedness of pain to OUD, a majority of participants agreed or strongly agreed that they first started using opioids due to pain (79, 56.4%), agreed or strongly agreed that pain is a major reason for their continued opioid use (76, 54.3%), confirmed having put off seeking treatment for OUD due to fear of pain exacerbation upon abstaining from opioids (81, 57.9%), and had a previous relapse experience in which pain was the direct motivator (82, 58.6%). These data are summarized in Figure 1.

### Correlational Analyses

Correlational analyses revealed relationships between pain severity and pain interference with perceptions that pain is related to OUD onset, maintenance, relapse, and treatment delay. For all correlative analyses, age and gender were mathematically controlled for via the use of nonparametric partial correlation as referenced in the analytical methods.

Constructs such as pain severity, pain interference, and the two pain interference dimensions exhibited an inherent degree of overlap. Though prior works support the notion that it is appropriate to view such constructs separately, it is still important to describe the landscape of relatedness between the constructs within the present sample. Table 2 is a brief correlation matrix between pain severity, total pain interference, affective pain interference, and activity pain interference.

**Table 2.** Spearman’s rho nonparametric partial correlation coefficients between pain severity, pain interference, affective pain interference, and activity pain interference in sample of individuals with untreated OUD.

	Pain severity	Total pain interference	Affective pain interference	Activity pain interference
Pain severity	1			
Total pain interference	0.702	1		
Affective pain interference	0.667	0.961	1	
Activity pain interference	0.674	0.946	0.825	1

Like all correlations of the present study, age and gender were controlled for when assessing correlations between pain constructs.

Pain severity scores in terms of average pain experienced over the past 4 weeks showed a moderate and statistically significant association with pain being the initial motivator for opioid use (*r_s_* [137] = 0.338, *P* = *<* 0.001, *P*_sig_ < 0.05), as well as pain being the primary reason for continued use (*r_s_* [137] = 0.444, *P* = *<* 0.001, *P*_sig_ < 0.05), and with pain having been the primary motivator for a past relapse experience (*r_s_* [137] = 0.371, *P* = *<* 0.001, *P*_sig_ < 0.05). Pain severity scores were also associated with putting off treatment for OUD due to fear of pain exacerbation upon abstinence from opioids (*r_s_* [137] = 0.298, *P* = *<* 0.001, *P*_sig_ < 0.05).

Relatedly, BPI pain interference totals displayed a moderate and statistically significant association with pain being a major reason for sustained opioid use (*r_s_* [137] = 0.403, *P* = *<* 0.001, *P*_sig_ < 0.05), as well as having experienced a pain-motivated relapse (*r_s_* [137] = 0.344, *P* = *<* 0.001, *P*_sig_ < 0.05). Pain interference totals also showed an association with pain being the initial motivator for opioid use (*r_s_* [137] = 0.274, *P* = 0.001, *P*_sig_ < 0.05), endorsing the injection of more opioids in an effort to control pain (*r_s_* [137] = 0.251, *P* = 0.003, *P*_sig_ < 0.05), and delaying treatment due to fear of pain exacerbation (*r_s_* [137] = 0.260, *P* = 0.002, *P*_sig_ < 0.05). Though the affective dimension of pain interference tended to display higher correlation coefficients than did the activity dimension, Fisher’s *Z* transformation analyses revealed that the affective and activity domain correlation coefficients did not significantly differ for any of the above relations, indicating that the pain interference totals were statistically representative of both domains with respect to the above series of correlations.

## Discussion

This study provides insight into how individuals with untreated OUD perceive pain and the relatedness of pain to OUD onset, maintenance, relapse, and treatment delay. The majority of participants agreed that pain was the reason they first began using opioids, pain motivated their ongoing opioid use, and fear of increased pain upon abstinence from opioids had caused them to delay OUD treatment. Nearly 60% of participants reported that they had previously been in remission and relapsed due to pain. Correlative analyses revealed that pain severity and pain interference were associated with perceptions that pain was related to OUD onset, maintenance, relapse, and treatment delay. Together, these findings suggest that pain may have an important role in the recalcitrance of OUD among non-treatment-seeking individuals.

Little prior research has examined pain or pain-motivated opioid use among non-treatment-seeking individuals with OUD. One study of 86 non-treatment-seeking individuals with OUD found that pain was present in 62% of the sample, pain severity and pain interference were elevated relative to controls without OUD, and participants reported that pain motivated their opioid use.^11^ However, that investigation included only individuals misusing prescription opioid analgesics and did not probe specific aspects of OUD relative to these pain measures. Thus, the present study expands such work by replicating these findings in a larger community sample with more diverse patterns of opioid use and demonstrating associations between these measures with extent of perceived relatedness of pain to individual aspects of OUD.

Eighty percent of participants in the present study reported having comorbid chronic pain. The prevalence of pain among non-treatment-seeking individuals with OUD is unknown. If pain is similarly pervasive among this population, there may be as many as 1.8 million people with untreated OUD who experience comorbid pain.^10^ However, the present study still has limitations on generalizability. Individuals who engage with substance use–related harm reduction services, such as a syringe exchange service, may not be fully representative of the broader population of individuals with untreated OUD. Nonetheless, the present work offers a partial enhancement in the generalizability of prior works. A greater understanding of pain and its perceived relatedness to OUD among non-treatment-seeking individuals is still needed. For example, the present study only looked at current treatment status and did not explore how treatment history may influence such perceptions. The overlap observed between pain interference domains also warrants caution in interpreting results. Further investigations might lead to new insights to improve OUD treatment engagement, receptivity, and outcomes.

Limited evidence informs the effective management of comorbid pain and OUD.^22,23^ Medications for OUD such as buprenorphine and methadone have collateral analgesic effects. However, the use of medications for OUD to treat pain in the setting of OUD is supported by mixed and generally low-quality evidence.^24^ Gauging the extent to which pain motivates opioid use in OUD, if employed alongside interference measures and more precise pain phenotyping, may be helpful in personalizing such treatment approaches. Our group has previously investigated central sensitization, a key mechanism of chronic pain, in the context of treatment-engaged individuals with OUD and yielded similar results regarding associations with endorsement of pain as a reason for OUD onset, maintenance, and relapse.^8^ Exploring whether particular pain mechanisms are found to have differential relationships with aspects of OUD and pain interference might facilitate the development of more targeted intervention strategies. The overlap between the activity and affective pain interference domains suggests that effective interventions should ideally work to target both domains of pain interference. However, the results of the present study do not allow us to deduce the complexities of such relationships, and the potential utility of individual variability between domains remains a subject of further research. Further research in such regards may be especially relevant to OUD as a chronic health condition marked by negative affect and chronic pain.^22,25^ Psychological treatments such as cognitive behavioral therapy and mindfulness have been suggested as modalities to improve negative affect and pain among individuals with pain and OUD.^23,26^ Exploring such notions further may lead to improved personalization of OUD treatment approaches. However, randomized controlled trials of these interventions have yet to show improvements in pain over standard care, and more research is needed.^27^ Nonetheless, the present study shows that pain is perceived to be important for OUD onset, maintenance, relapse, and treatment delay among non-treatment-seeking individuals with OUD, and these perceptions are associated with measures of pain severity and pain interference.

## Conclusion

This study described pain and its perceived relatedness to OUD among individuals with untreated OUD. A majority of participants agreed that pain was the reason their OUD began, that pain was the reason they continued to use opioids, and that fear of worsening pain has caused them to delay OUD treatment. Greater pain severity and pain interference were associated with greater agreement with these statements. Future studies should seek to determine whether prioritizing pain management might improve OUD treatment engagement and receptivity among non-treatment-seeking individuals with comorbid pain and OUD. Future investigations should also work to explore relationships between aspects of OUD and specific types of pain in terms of features such as underlying cause, phenotype, and mechanism.
